# Editorial: Neuropsychiatric symptoms and cognitive impairment

**DOI:** 10.3389/fneur.2025.1680162

**Published:** 2025-10-01

**Authors:** Ioannis Liampas, Efthimios Dardiotis, Vasileios Siokas, Robert Fleischmann

**Affiliations:** ^1^Department of Neurology, Faculty of Medicine, School of Medicine, University of Thessaly, Mezourlo Hill, Larissa, Greece; ^2^Department of Neurology, University Medicine Greifswald, Greifswald, Germany

**Keywords:** dementia, cognitive impairment, neuropsychiatric symptoms, inappropriate trusting behavior, mild cognitive impairment

Neuropsychiatric symptoms (NPS) are increasingly recognized not as mere consequences of cognitive decline, but as core features of minor and major neurocognitive entities. Mounting evidence suggests that affective symptoms, emotional lability, behavioral changes and/or psychotic symptoms may precede cognitive deficits (1, 2), predict disease progression (3, 4), institutionalization, and mortality (5), profoundly affect patients' quality of life and caregiver burden (6). Yet, clinical and research paradigms often maintain a fragmented view, separating cognition from behavior and emotion and thus limiting diagnostic precision and impeding holistic interventions. This fragmentation is further compounded by the prevailing reliance on observable phenotypes rather than on pathophysiologically grounded endotypes. Disorders like delirium or dementia are still largely defined by surface-level behavioral and cognitive criteria, despite emerging evidence that distinct or overlapping biological mechanisms, i.e., endotypes, may underlie superficially similar presentations (7). The conflation or divergence of phenotypes and endotypes introduces substantial bias into clinical research and mechanistic modeling, particularly when converging symptoms mask heterogeneous etiologies or when similar biological processes present with diverging behavioral features (8). Integrating biological signatures into diagnostic frameworks could therefore sharpen our understanding of progression, prognosis, and treatment response.

This Research Topic was conceived to address that gap by focusing on the interplay between NPS and cognitive impairment, while also incorporating approaches such as neuroimaging and fluid biomarkers to overcome the limitations of purely phenotypic classification to move toward biologically grounded endotypes. Some authors examined the understudied dimensions of positive affect and social vulnerability, others explored caregiver burden and finally some studies focused on non-medicinal interventions and their merit in the management of cognitive and/or behavioral symptoms in older adults with cognitive impairment. The 16 articles published draw from heterogeneous clinical populations across different continents. They span a rich diversity of methodologies and perspectives, including epidemiological analyses, systematic reviews and network meta-analyses, machine learning approaches, protocol development, and bibliometric mapping. This editorial synthesizes the key contributions of these studies and outlines how they enhance published literature and advance an integrative model of cognitive health—one in which *Neuropsychiatric symptoms and cognitive impairment* are seen as core, interconnected elements of minor and major neurocognitive disorders.

First, Chokesuwattanaskul et al. explored a relatively underexplored behavioral dimension in dementia: inappropriate trusting behavior. Inappropriate trusting was more common in semantic variant primary progressive aphasia (svPPA, 55%) and behavioral variant FTD (bvFTD, 44%), than non-fluent/agrammatic variant primary progressive aphasia (nfvPPA, 17%) and AD (24%).The authors identified neuropsychiatric, cognitive, and psychosocial predictors of such behavior across different dementia syndromes; apathy in the semantic variant primary progressive aphasia (PPA), disinhibition and altered pain responsiveness in the behavioral variant FTD, and lower mini-mental state examination (MMSE) and revised self-monitoring scale (RSMS) scores in AD. Such findings offer new insights into social vulnerability and ethical care, with implications for clinical counseling and safeguarding strategies for patients with impaired judgment.

While the vast majority of studies on NPS focus on identifying and treating “negative emotion,” Miklitz et al. argue for a more holistic understanding of neuropsychiatric health in dementia. Their work emphasizes positive affect, happiness, and subjective wellbeing; dimensions often overlooked in dementia care. The authors argue that due to the profound cognitive impairment, past events and future thinking assume less important roles in the subjective wellbeing of patients with dementia and momentary experiencing becomes more significant. They propose the study of positive emotions in the experienced moment of the participants using ecological momentary assessments. By highlighting emotional resilience and the potential for wellbeing even in the context of cognitive decline, they challenge negative emotion-oriented paradigms and offer direction for future research and dementia trials.

Next, the role of NPS as prodromal signs of cognitive impairment were explored. Yakemow et al. applied machine learning to MRI and clinical data in individuals with post-traumatic stress disorder (PTSD). The authors identified neuroimaging patterns emulating those seen in early Alzheimer's disease (AD). Their findings suggest that PTSD-related NPS paired with structural brain changes may serve as early indicators of an increased risk for future development of dementia. Peixoto et al. extended this early-detection focus to the post-COVID-19 context. They investigated cognitive performance 6 months after infection with the SARS-CoV-2 gamma variant. Their findings identified anxiety symptoms, among others, as key correlates of persistent cognitive impairment. This underscores the relevance of neuropsychiatric symptomatology in post-viral cognitive syndromes, especially in older adults. Finally, Rao et al. conducted comprehensive neuropsychological assessments in a community-based Chinese cohort, revealing a high prevalence (16%) of amnestic mild cognitive impairment (aMCI) in individuals aged 60 and above. Sleep disorders were not related to aMCI, which is a matter of ongoing debate in literature (9). Memory, language and executive impairments were more predominant, with long-term delayed recall emerging as the strongest predictor of aMCI. These findings reinforce the clinical importance of targeted memory assessments, particularly for verbal learning and retention, in early detection.

The biological underpinnings of cognitive impairment were examined through neuroimaging and metabolic frameworks. Mu et al. demonstrated that individuals with vascular cognitive impairment due to cerebral small vessel disease showed aberrant resting-state brain activity and disrupted network connectivity, correlating with specific domains of cognitive dysfunction. Their results point to neurofunctional mechanisms underlying cognitive disturbances in vascular dementia. Ye et al. conducted a dose–response meta-analysis on hypoglycemic events in individuals with type 2 diabetes mellitus. Their findings demonstrated a significant relationship between the frequency of hypoglycemia and the risk of cognitive decline, reinforcing the neurocognitive vulnerability conferred by metabolic stress. Liu et al. complemented this by assessing the prevalence of cognitive dysfunction in diabetic populations and the potential for tailored treatment strategies. Together, these studies highlight a metabolic–neurocognitive interplay in aging that merits further exploration. Long et al. conducted a systematic review and meta-analysis examining the role of trimethylamine-N-oxide (TMAO), a gut-derived metabolite. Elevated TMAO levels were associated with cognitive impairment, linking peripheral metabolic signals to cognitive dysfunction. These findings lend support to emerging gut–brain interaction models in dementia research.

This Topic includes several studies evaluating non-pharmacological interventions for cognitive and neuropsychiatric outcomes. Cai et al. focused specifically on the use of exergames—digitally mediated physical activities—reporting significant improvements in cognitive function among older adults with MCI. Previous research has also demonstrated that exergames may improve NPS in neurocognitive disorders (10). Overall, these interventions offer scalable, engaging strategies for dual cognitive and neuropsychiatric benefits. Chen and Kim systematically reviewed the impact of mind–body and aerobic exercise programs on individuals with MCI. They reported small to moderate benefits in reducing depressive symptoms and improving quality of life—findings that support the utility of lifestyle-based interventions in early-stage cognitive impairment. In a complementary bibliometric analysis, Zhang et al. mapped the global research landscape on body–mind exercises in MCI. Their analysis highlights the growing interdisciplinary interest in interventions that simultaneously target physical, emotional, and cognitive health. Yi et al. synthesized existing data on non-pharmacological adjunctive therapies for vascular dementia (e.g., acupuncture, repetitive transcranial magnetic stimulation, and so on) through network meta-analysis. They found modest but consistent effects on both cognitive performance and functional outcomes, providing actionable insights for evidence-based dementia care planning. Arrieta et al. detailed the FORTCARE-MCI study protocol, which evaluates Fortasyn Connect in individuals with MCI in primary care settings. Fortasyn Connect is a specialized medical food combining nutrients formulated to support memory function in early AD. Importantly, the trial includes both cognitive and behavioral outcome measures, reflecting a translational emphasis on real-world effectiveness and patient-centered evaluation.

Huang et al. presented a striking case of Creutzfeldt–Jakob disease that was initially misdiagnosed as a primary psychiatric condition. Their case review underscores the importance of maintaining diagnostic suspicion when atypical behavioral symptoms appear late in life. This holds particular significance especially when they progress rapidly or are unresponsive to conventional psychiatric treatment.

Finally, Li et al. evaluated caregivers of individuals with Alzheimer's disease across different disease stages. They reported that patients' mental states substantially influenced caregiver distress and psychological health. These conclusions reinforce the bidirectional burden of dementia and the necessity of psychosocial support in family-based care.

Together, the studies in this Research Topic affirm that neuropsychiatric symptoms are core features integral—not incidental—to cognitive disorders (11). They are early markers of pathology, contributors to functional impairment, drivers of caregiver distress, and potential treatment targets (12, 13). Integrating NPS into the clinical and research frameworks of neurodegeneration allows for more precise diagnosis, earlier management and more holistic care (14). Moreover, NPS can offer unique pathophysiological insights into the underlying disease, as they often manifest during critical states of network dysfunction (15). In such states, neurobiological alterations may become visible that would otherwise remain undetected under stable or compensated conditions. Without acknowledging the relevance of NPS within cognitive disorders, biologically meaningful signatures and potential endotypes risk being overlooked. This body of work reflects the strength of interdisciplinary research in shaping the future of neurocognitive health. As editors, we hope that the findings presented here will guide further work in developing integrative, individualized models of dementia prevention and care.

## Generative AI statement

The author(s) declare that no Gen AI was used in the creation of this manuscript.

Any alternative text (alt text) provided alongside figures in this article has been generated by Frontiers with the support of artificial intelligence and reasonable efforts have been made to ensure accuracy, including review by the authors wherever possible. If you identify any issues, please contact us.
